# Circular RNA circUBAP2 regulates proliferation and invasion of osteosarcoma cells through miR-641/YAP1 axis

**DOI:** 10.1186/s12935-020-01318-4

**Published:** 2020-06-08

**Authors:** Haojie Wu, Weihua Li, Shutao Zhu, Dengfeng Zhang, Minghui Zhang

**Affiliations:** Department of Orthopaedics, Huaihe Hospital, Henan University, No. 8 Baobei Road, Gulou District, 475001 Kaifeng, Henan China

**Keywords:** OS, circUBAP2, miR-641, YAP1, Proliferation, Invasion

## Abstract

**Background:**

Osteosarcoma (OS) is a common malignant bone cancer and is still a growing threat to young people. Circular RNAs (CircRNAs) are reported to be involved in the development of diverse human cancers. However, the role of circUBAP2 in OS progression is rarely reported.

**Methods:**

Quantitative real-time polymerase chain reaction (qRT-PCR) was conducted to detect the expression levels of circUBAP2 and miR-641 in OS tissues and cells. Cell Counting Kit-8 (CCK-8) assay was employed to check cell proliferation. The ability of cell invasion was evaluated by transwell assay. The protein levels of E-cadherin, Vimentin and Yes-associated protein 1 (YAP1) were measured by western blot. The starBase was used to predict binding sites between miR-641 and circUBAP2 or YAP1 and the dual-luciferase reporter assay was performed to verify the interaction.

**Results:**

The level of circUBAP2 was significantly upregulated in OS tissues and cells compared with normal tissues and cells, which was contrary to the expression of miR-641. Downregulation of circUBAP2 suppressed proliferation and invasion of OS cells and weakened the process of epithelial-mesenchymal transition (EMT). Moreover, miR-641 was a target of circUBAP2 and could bind to the 3′-untranslated region (3′UTR) of YAP1. In addition, overexpression of circUBAP2 or YAP1 reversed the inhibitory effects of miR-641 on proliferation and invasion of OS cells. Further research indicated that circUBAP2 regulated the expression of YAP1 by interacting with miR-641 in OS cells.

**Conclusion:**

Knockdown of circUBAP2 impeded proliferation and invasion of OS cells by downregulating the expression of YAP1 via sponging miR-641.

## Highlights

1. circUBAP2 was upregulated in osteosarcoma tissues and cells.
2. Downregulation of circUBAP2 inhibited proliferation and invasion of osteosarcoma cells.
3. circUBAP2 was a target of miR-641 and its expression was negatively correlated with miR-641.
4. MiR-641 bound to the 3′UTR of YAP1 and negatively modulated the expression of YAP1.
5. circUBAP2 regulated YAP1 expression by sponging miR-641 in osteosarcoma cells.

## Background

Osteosarcoma (OS) develops in the skeletal system and can spread to distant organs, mainly occurring in teenagers and young adults [1]. Although the survival rate has been greatly elevated in recent years, some patients with clinical metastasis have a poor prognosis and their 5-year survival rates are not more than 30% [2]. Hence, it is pressing to find novel molecular targets for OS treatment.

Circular RNAs (CircRNAs), a type of single-stranded RNA which forms a covalently closed continuous loop, are produced by backsplicing [3] and they are resistant to exonuclease-mediated degradation [4]. CircRNAs are reported to be associated with lung adenocarcinoma [5], colon cancer [6], hepatocellular carcinoma [7] and gastric cancer [8], as well as OS [9]. Zhang et al. found that circUBAP2 was highly expressed in OS tissues and inhibited cell apoptosis in vivo [10]. Yet, the regulatory mechanism of circUBAP2 in OS progression has not been fully addressed. Moreover, circular RNAs are known to enhance intercellular adhesion molecule 1 (ICAM-1) [11] and metastatic cancerous cells also have more C3aR, the receptor of complement fragment C3a [12].

MicroRNAs (MiRNAs) are short (about 22 nucleotides) and highly conserved noncoding RNAs, which modulate gene expression by binding to the 3′-untranslated region (3′UTR) of messenger RNA (mRNA) at the post-transcriptional level [13]. Growing papers have emphasized the core position of miRNAs in controlling cancer development [14–16]. MiR-641 has been reported to be dysregulated in many human cancers [17, 18] and a recent report indicated that miR-641 was downregulated in OS tissues [19]. Nevertheless, the function of miR-641 in OS development needs to be further explored.

Yes-associated protein 1 (YAP1), also known as YAP or YAP65, is a transcriptional co-activator [20] and is involved in lots of cancer progression. Thompson et al. found that YAP1 promoted cutaneous squamous cell carcinoma formation and progression [21]. Yi et al. confirmed that YAP/TAZ signaling was associated with tumor immune evasion [22]. Also, Shen et al. reported that overexpression of YAP1 inhibited anoikis in osteosarcoma cells [23]. Therefore, YAP1 may be an appealing drug target for OS and it is worth intensely studying.

In this research, the expression level of circUBAP2 in OS tissues and cells was checked. The function and potential regulatory mechanism of circUBAP2 in OS were further investigated by bioinformatic analysis and subsequent experiments.

## Materials and methods

### Samples and cell culture

OS tissues and nearby non-cancerous tissues were collected from Huaihe Hospital, Henan University. The informed consent was acquired from every patient and this study was authorized by the Ethics Committee of Huaihe Hospital, Henan University. Human osteoblastic cell line (hFOB1.19) and OS cell lines (U2OS and SaOS2) were obtained from American Type Culture Collection (ATCC, Manassas, VA, USA). McCoy’s 5A medium (Sigma, St Louis, MO, USA), containing 5% CO_2_ and 10% fetal bovine serum (FBS) (Sigma), was utilized to culture cells.

### Cell transfection

Small interfering RNA against circUBAP2 (named as si-circUBAP2), miR-641 mimic (named as miR-641), miR-641 inhibitor (named as Anti-miR), as well as their corresponding controls, were obtained from GenePharma (Shanghai, China). circUBAP2 expression plasmid (named as OE-circUBAP2), YAP1 expression plasmid (named as OE-YAP1) and matched control (named as Vector) were acquired from RiboBio (Guangzhou, China). Cell transfection was performed using Lipofectamine 2000 reagent (Invitrogen, Carlsbad, CA, USA) following the provided procedures.

### RNA isolation and quantitative real-time polymerase chain reaction (qRT-PCR)

OS tissues and cells were collected and total RNA was extracted using the TRIzol reagent (Vazyme, Nanjing, China). Then RNA was reversely transcribed to complementary DNA (cDNA) by PrimeScript™ RT Master Mix kit (Takara, Dalian, China). The qRT-PCR was performed by SYBR Green PCR Master Mix (Vazyme) and data were analyzed using 2^−ΔΔCt^ method. Beta-actin (β-actin) and U6 were introduced as the inner references. Primers in this research:

circUBAP2 (forward 5′-AGCCTCAGAAGCCAACTCCTTTG-3′, reverse 5′-TCAGGTTGAGATTTGAAGTCAAGAT-3′); miR-641 (forward, 5′-GGGGAAAGACATAGGATAGAGT-3′, reverse 5′-CAGTGCGTGTCGTGGAG-3′); YAP1 (forward, 5′-CCTGATGGATGGGAACAAGC-3′, reverse 5′-GCACTCTGACTGATTCTCTGG-3′); β-actin (forward 5′-GCACCACACCTTCTACAATG-3′, reverse, 5′-TGCTTGCTGATCCACATCTG-3′); U6 (forward, 5′-TCCGGGTGATGCTTTTCCTAG-3′, reverse, 5′-CGCTTCACGAATTTGCGTGTCAT-3′).

### Counting Kit-8 (CCK-8) assay

After transfection, U2OS and SaOS2 cells were seeded into 96-well plates and then incubated with 10 μL CCK-8 solution (Beyotime, Shanghai, China) for 2 h. Optical density values were examined at 450 nm wavelength under the microplate reader (Bio-Rad, Richmond, Virginia, USA).

### Transwell assay

Transwell chamber precoated with Matrigel (Solarbio, Beijing, China) was utilized to check the ability of cell invasion. Transfected cells were seeded into the upper chamber and medium containing FBS was placed into the lower chamber. After being treated with crystal violet (Solarbio), invaded cells were analyzed under an inverted microscope (MTX Lab Systems, Bradenton, FL, USA).

### Western blot

Proteins from cells were isolated using RIPA buffer (Vazyme) and the concentration was checked by Detergent Compatible Bradford Protein Quantification Kit (Vazyme). Subsequently, sodium dodecyl sulfate polyacrylamide gel electrophoresis (SDS-PAGE) was used to segregate proteins and then the proteins were transferred onto the polyvinylidene difluoride (PVDF) membranes (Vazyme). After being blocked with 5% skimmed milk (Vazyme) and washed by phosphate-buffered saline (PBS), the membranes were incubated with the primary antibodies: E-cadherin (1:3000, ab40772, Abcam, Cambridge, United Kingdom), Vimentin (1:2500, ab92547, Abcam), YAP1 (1:3000, ab52771, Abcam), C3aR (1:1000, ab126250, Abcam), ICAM-1 (1:1000, ab2213, Abcam), or β-actin (1:2500, ab8227, Abcam) overnight. After being rewashed with PBS, the membranes were incubated with the secondary antibody (1:3000, ab205718, Abcam) for 3 h. The membranes were analyzed by the ChemiDoc™ MP Imaging System (Bio-Rad, Richmond, CA, USA) after being treated with ECL kit (Vazyme).

### Dual-luciferase reporter assay

The potential complementary sequences between miR-641 and circUBAP2 or YAP1 were forecasted by starBase [24]. The wild type sequence of circUBAP2 or YAP1 3′UTR harboring the target sites of miR-641 was inserted to the pGL3 vector (ShengZhaobio, Shanghai, China) to construct the luciferase reporter vector circUBAP2 WT or YAP1-WT, respectively. Similarly, circUBAP2 MUT or YAP1-MUT was established by mutating the potential target sites of miR-641, respectively. Then, the vectors with miR-641 or miR-NC were cotransfected into U2OS and SaOS2 cells using Lipofectamine 2000 (Invitrogen). The Dual-Glo^®^ Luciferase Assay System kit (Promega) was utilized to measure luciferase activity.

### Statistical analysis

Experimental data were calculated by GraphPad Prism (GraphPad, La Jolla, CA, USA) and presented by mean ± standard deviation (SD). Two independent groups were compared by using Student’s *t* test. For more than two groups, the one-way analysis of variance (ANOVA) was utilized to evaluate the difference. Pearson’s correlation coefficient was utilized to analyze the correlation between circUBAP2 and miR-641 in OS tissues. Every experiment was repeated at least three times independently. *P* < 0.05 represented statistical significance.

## Results

### circUBAP2 was significantly upregulated in OS tissues and cells

**Fig. 1:**
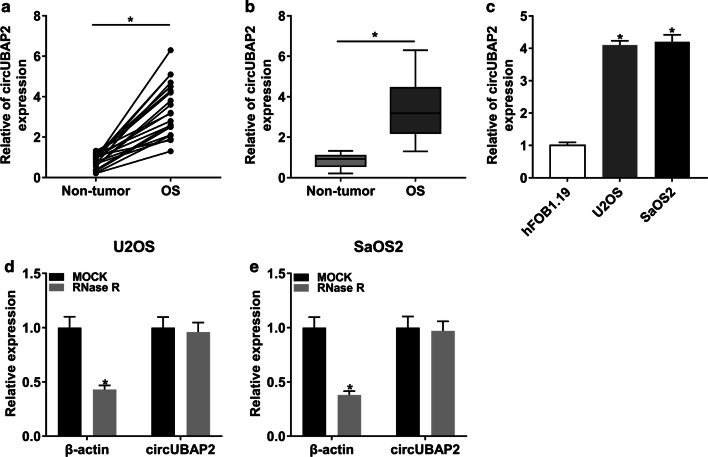
circUBAP2 was significantly upregulated in OS tissues and cells. **a**, **b** The expression level of circUBAP2 in normal tissues and tumor tissues was detected by qRT-PCR. **c** The expression level of circUBAP2 in normal cells and OS cells was checked by qRT-PCR. **d**, **e** The relative expression of β-actin and circUBAP2 in OS cells treated with RNase R or not was measured by qRT-PCR. *P < 0.05

To explore the role of circUBAP2, we first checked its expression level in OS tissues and cells. The results showed that circUBAP2 was significantly upregulated in OS tissues and cells compared with corresponding controls (Fig. 1a–c). Besides, we also found that the level of circUBAP2 in OS cells was not notably changed under the treatment of RNase R, indicating that circUBAP2 was resistant to RNase R (Fig. 1d, e). These results disclosed that circUBAP2 might act as an oncogene in OS.

### Knockdown of circUBAP2 repressed proliferation and invasion of OS cells

**Fig. 2:**
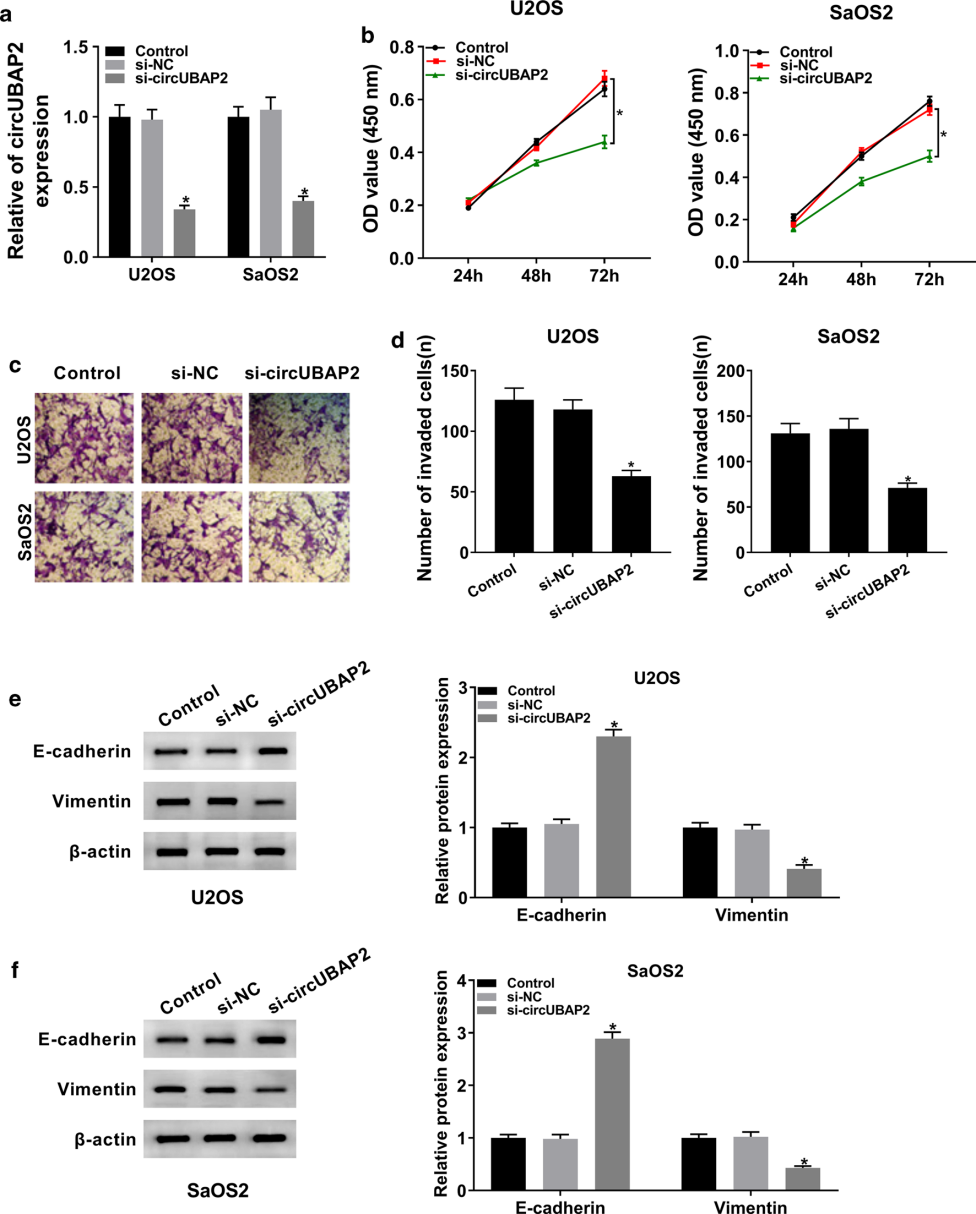
Knockdown of circUBAP2 hampered proliferation and invasion of OS cells. **a** The expression of circUBAP2 in OS cells transfected with si-circUBAP2, as well as matched controls, was assessed by qRT-PCR. **b** Cell proliferation at different time points was evaluated by CCK-8 assay. **c**, **d** Transwell assay was utilized to check cell invasion, and corresponding invaded cells were calculated. **e**, **f** The protein levels of E-cadherin and Vimentin in Control and transfected OS cells were determined by western blot. *P < 0.05

To further explore the function of circUBAP2 in OS, we checked its expression in OS cells transfected with si-circUBAP2, as well as matched controls. The data showed that circUBAP2 was conspicuously decreased in si-circUBAP2 group compared with si-NC group or Control group (Fig. 2a). Next, CCK-8 assay and transwell assay were carried out to study the role of circUBAP2 in OS cell proliferation and invasion, respectively. CCK-8 assay indicated that downregulation of circUBAP2 strikingly hindered proliferation of OS cells (Fig. 2b). Transwell assay showed that the ability of invasion of U2OS and SaOS2 cells was apparently weakened in si-circUBAP2 group (Fig. 2c, d). Moreover, the protein levels of E-cadherin and Vimentin in OS cells were measured and the results showed that knockdown of circUBAP2 markedly increased the expression of E-cadherin while downregulated Vimentin (Fig. 2e, f). Besides, our data showed that circUBAP2 knockdown decreased the expression of C3aR and ICAM-1 in both U2SO and SaOS2 cells (Additional file 1: Figure S1A and B). Collectively, these results demonstrated that circUBAP2 silencing could inhibit proliferation and invasion of OS cells in vitro.

### circUBAP2 was a target of miR-641 and negatively regulated the expression of miR-641 in OS cells

**Fig. 3:**
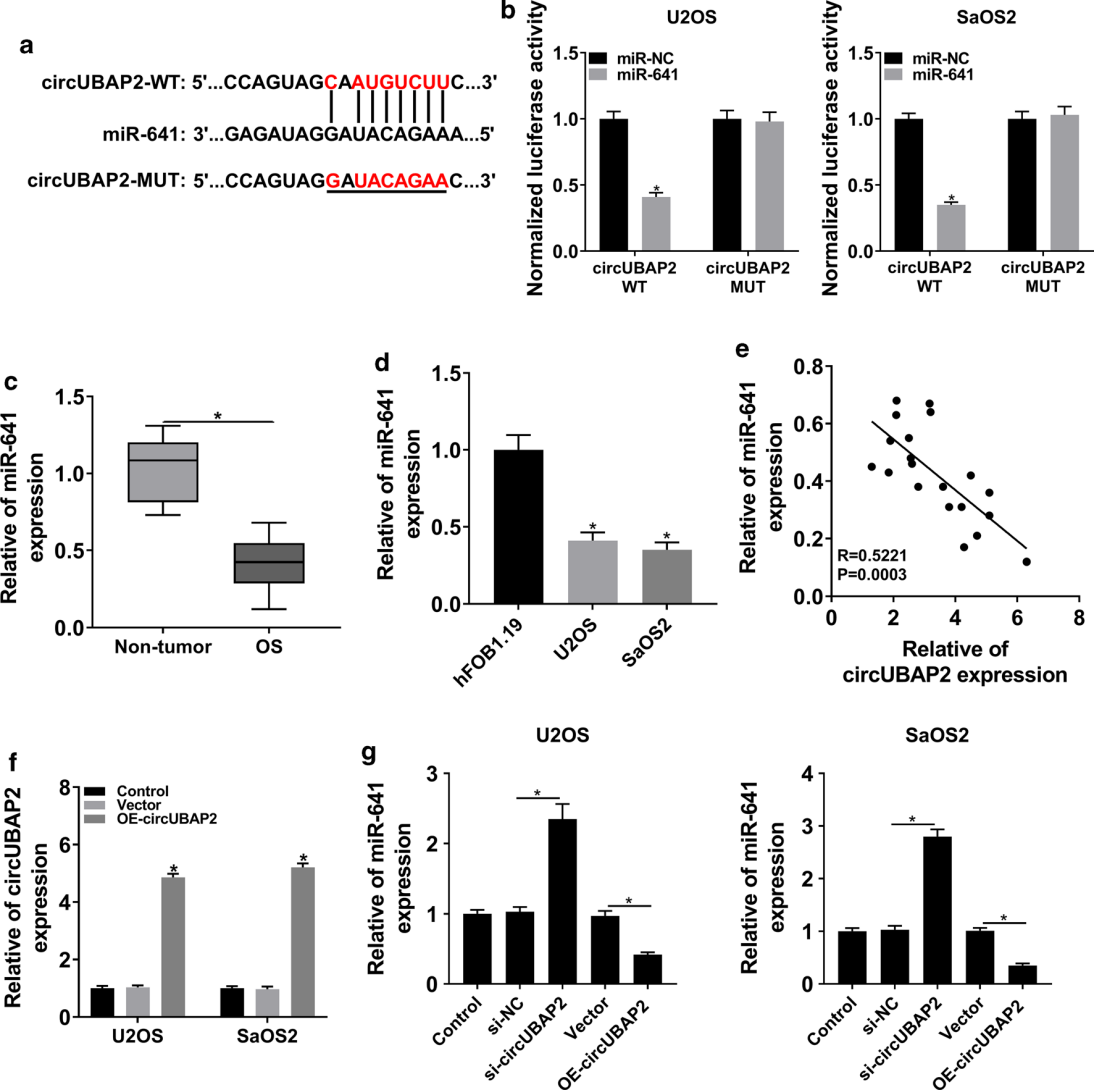
circUBAP2 interacted with and negatively regulated miR-641 in OS cells. **a** The putative binding sites between circUBAP2 and miR-641 were predicted by starBase. **b** The luciferase activity in OS cells cotransfected with miR-641 and circUBAP2 WT or circUBAP2 MUT was checked. **c**, **d** The level of miR-641 in OS tissues and cells was evaluated by qRT-PCR. **e** The correlation between circUBAP2 and miR-641 in OS tissues was determined using Pearson’s correlation coefficient. **f** The expression of circUBAP2 in OS cells transfected with OE-circUBAP2, as well as matched controls, was checked by qRT-PCR. **g** The level of miR-641 in Control and OS cells transfected with si-NC, si-circUBAP2, Vector or OE- circUBAP2 was measured by qRT-PCR. *P < 0.05

The interaction between circRNAs and miRNAs in cancers was documented in many reports [25, 26]. In this study, we found that circUBAP2 harbored the binding sites of miR-641 (Fig. 3a). To verify this interaction, the dual-luciferase reporter assay was performed and the results showed that miR-641 significantly diminished the luciferase activity of circUBAP2 WT in OS cells, rather than circUBAP2 MUT (Fig. 3b). We then checked the expression of miR-641 and the data indicated that miR-641 was markedly declined in OS tissues and cells (Fig. 3c, d). Correlation analysis elucidated that the expression of miR-641 was negatively associated with circUBAP2 in OS tissues (Fig. 3e). To figure out the regulatory relationship between the two in OS cells, circUBAP2 expression plasmid was constructed and the overexpression efficiency was tested by qRT-PCR (Fig. 3f). Afterwards, the expression of miR-641 in OS cells infected with si-circUBAP2 or OE-circUBAP2, as well as matched controls, was measured. The results showed that downregulation of circUBAP2 significantly increased the expression of miR-641, whereas overexpression of circUBAP2 significantly decreased the level of miR-641 (Fig. 3g). All in all, these results illustrated that circUBAP2 interacted with miR-641 and negatively modulated the expression of miR-641 in OS cells.

### MiR-641 impeded proliferation and invasion of OS cells in vitro

**Fig. 4:**
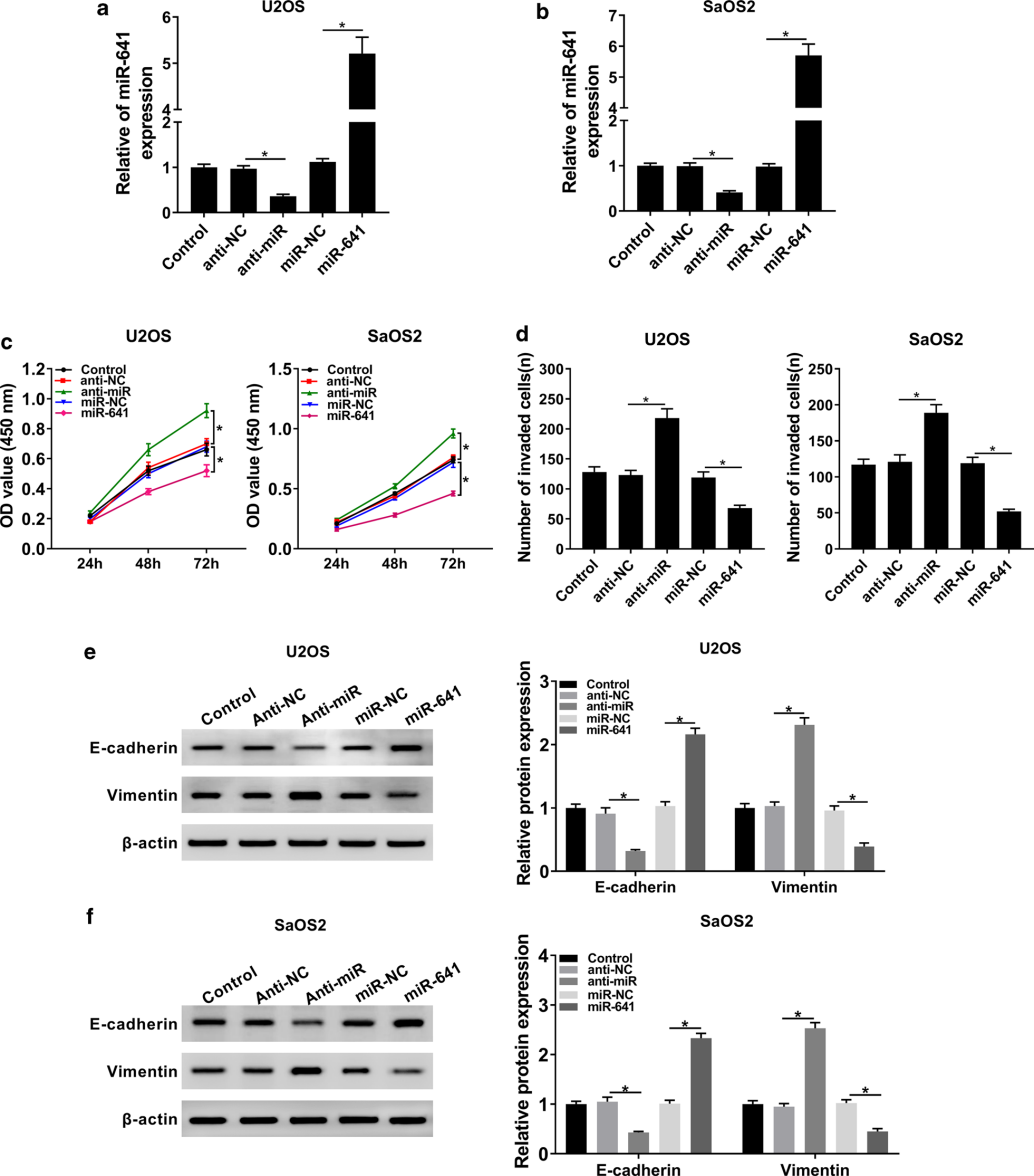
MiR-641 inhibited proliferation and invasion of OS cells. **a**, **b** The expression level of miR-641 in Control and OS cells transfected with Anti-miR, miR-641, or corresponding negative controls was checked by qRT-PCR. **c** Cell proliferation at different time points was assessed by CCK-8 assay. **d** Transwell assay was employed to evaluate the ability of cell invasion, and corresponding invaded cells were calculated. **e**, **f** The protein levels of E-cadherin and Vimentin in Control and transfected OS cells were checked by western blot. *P < 0.05

To investigate the function of miR-641 in OS, the level of miR-641 in OS cells transfected with Anti-miR or miR-641, as well as corresponding controls, was detected. The data showed that miR-641 was evidently downregulated in Anti-miR group, while its expression was clearly upregulated in miR-641 group (Fig. 4a, b). Besides, MTT assay indicated that miR-641 mimic conspicuously repressed proliferation of OS cells, while downregulation of miR-641 promoted proliferation of OS cells (Fig. 4c). Similarly, miR-641 mimic obstructed cell invasion while miR-641 inhibitor enhanced the ability of invasion of OS cells (Fig. 4d). Further studies manifested that downregulation of miR-641 reduced the level of E-cadherin and elevated the expression of Vimentin, while overexpression of miR-641 got the opposite results (Fig. 4e, f). Altogether, these results supported the idea that miR-641 might function as a tumor suppressor in OS progression.

### Overexpression of circUBAP2 rescued the inhibitory effects of miR-641 on proliferation and invasion of OS cells

**Fig. 5:**
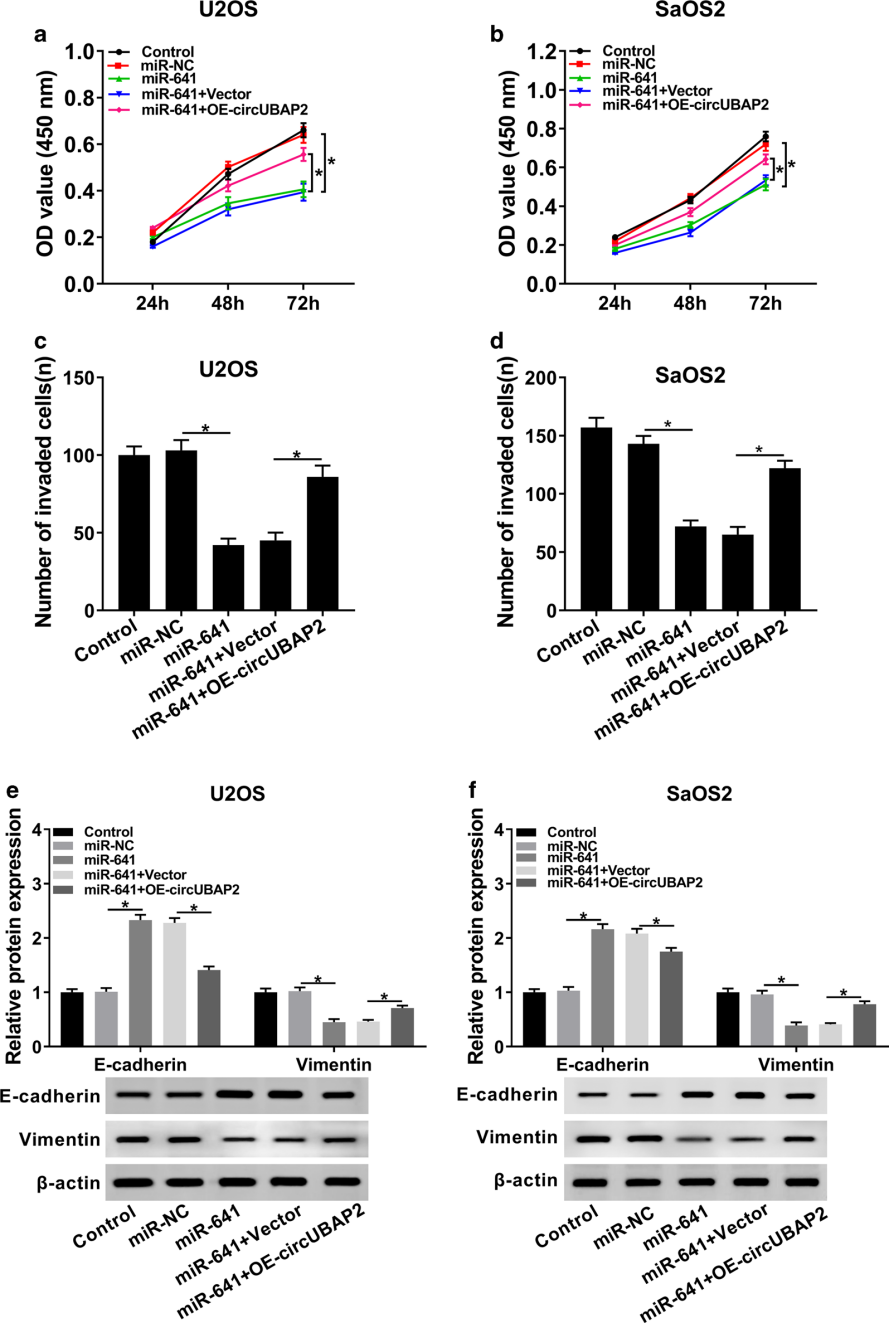
Overexpression of circUBAP2 inverted the effects of miR-641-mediated on proliferation and invasion of OS cells. **a**, **b** Cell proliferation at different time points was checked by CCK-8 assay. **c**, **d** The capability of cell invasion was evaluated by transwell assay, and the invaded cells were calculated. **e**, **f** The protein levels of E-cadherin and Vimentin in Control and transfected OS cells were evaluated by western blot. *P < 0.05

We further dissected the impacts of the interaction between circUBAP2 and miR-641 on proliferation and invasion of OS cells. MTT assay indicated that upregulation of circUBAP2 inverted miR-641-mediated repressed effect on proliferation of OS cells (Fig. 5a, b). Meanwhile, the effect of miR-641-mediated suppression on invasion was reversed by overexpression of circUBAP2 (Fig. 5c, d). In addition, the expression levels of E-cadherin and Vimentin in miR-641 group were clearly transposed after the transfection with OE-circUBAP2 (Fig. 5e, f). In summary, these results elucidated that miR-641 and circUBAP2 played opposite roles in OS progression, and enforced expression of circUBAP2 overturned the impacts of miR-641-mediated on proliferation and invasion of OS cells.

### YAP1 was a target of miR-641 and its overexpression inverted miR-641-mediated effects on proliferation and invasion of OS cells

**Fig. 6:**
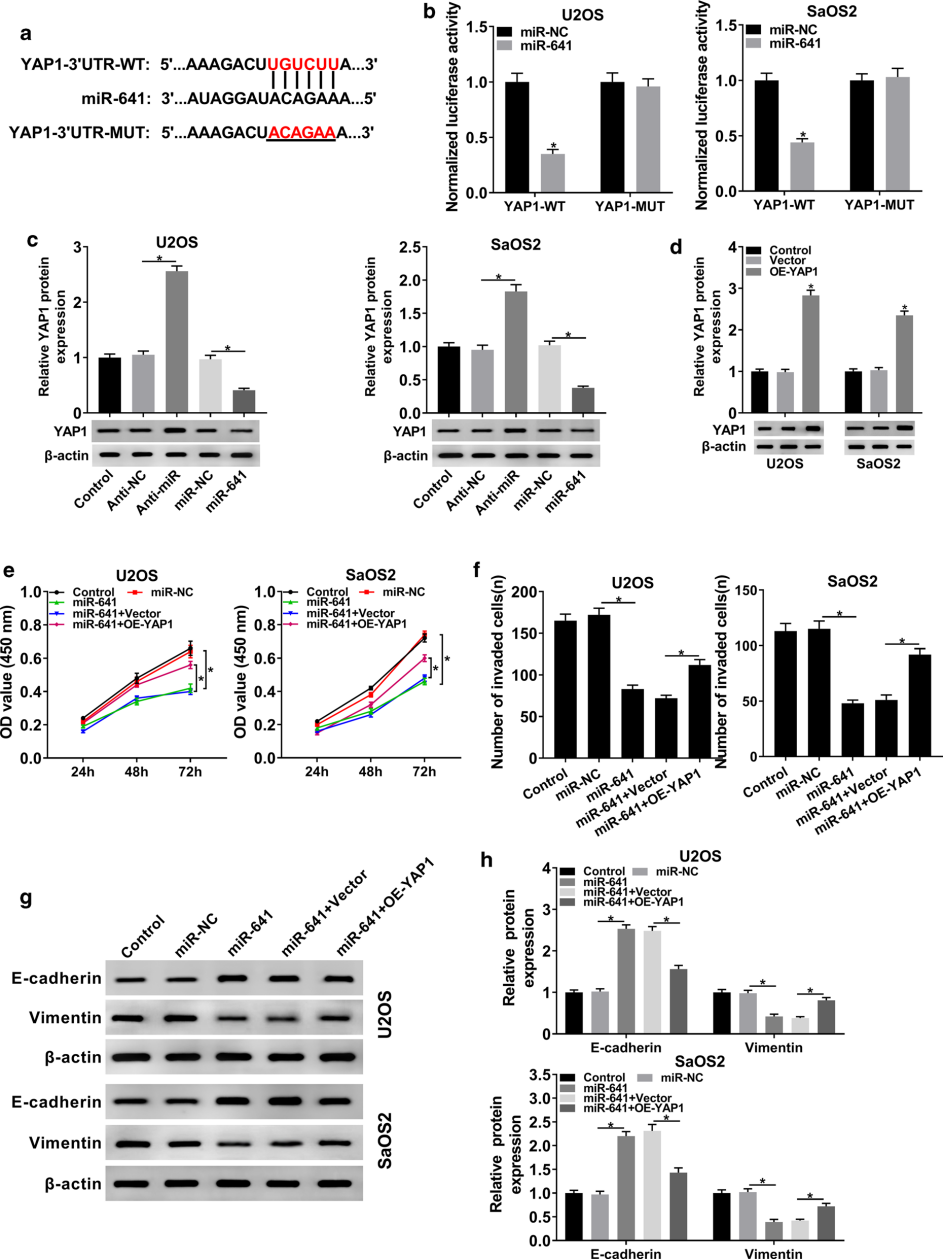
MiR-641 bound to the 3′UTR of YAP1 and its impacts on proliferation and invasion were transposed by YAP1 overexpression. **a** The putative target sites between miR-641 and YAP1 were predicted by starBase. **b** The dual-luciferase reporter assay was performed to corroborate the interaction between miR-641 and YAP1. **c** The protein level of YAP1 in Control and OS cells infected with Anti-miR, miR-641 or matched controls was detected by western blot. **d** The protein level of YAP1 in Control and OS cells infected with Vector or OE-YAP1 was measured by western blot. **e** Cell proliferation at different time points was checked by CCK-8 assay. **f** Transwell assay was hired to check cell invasion and corresponding invaded cells were counted. **g**, **h** The protein levels of E-cadherin and Vimentin in Control and infected OS cells were determined by western blot. *P < 0.05

To figure out the potential mechanism of miR-641 in OS, starBase was hired to find its possible target genes. The results indicated that miR-641 could bind to the 3′UTR of YAP1 (Fig. 6a), and this interaction was confirmed by the dual-luciferase reporter assay (Fig. 6b). Next, we checked the protein level of YAP1 in OS cells transfected with Anti-miR or miR-641, as well as corresponding controls. The results showed that downregulation of miR-641 notably elevated the expression of YAP1, whereas upregulation of miR-641 clearly decreased the level of YAP1 (Fig. 6c). To study the function of YAP1 in OS progression, overexpression plasmid of YAP1 was constructed and the efficiency was verified (Fig. 6d). MTT assay showed that overexpression of YAP1 reversed miR-641-mediated suppressed effect on proliferation of OS cells (Fig. 6e). Also, the inhibitory effect of miR-641 on invasion of OS cells was abolished by YAP1 overexpression (Fig. 6f). Similarly, the protein levels of E-cadherin and Vimentin in miR-641 group were reversely changed following the transfection with OE-YAP1 (Fig. 6g, h). From these results, it could be concluded that miR-641 negatively modulated YAP1 in OS cells and its repressed effects on proliferation and invasion of OS cells were inverted by upregulating YAP1.

### circUBAP2 regulated the expression of YAP1 by targeting miR-641 in OS cells

**Fig. 7:**
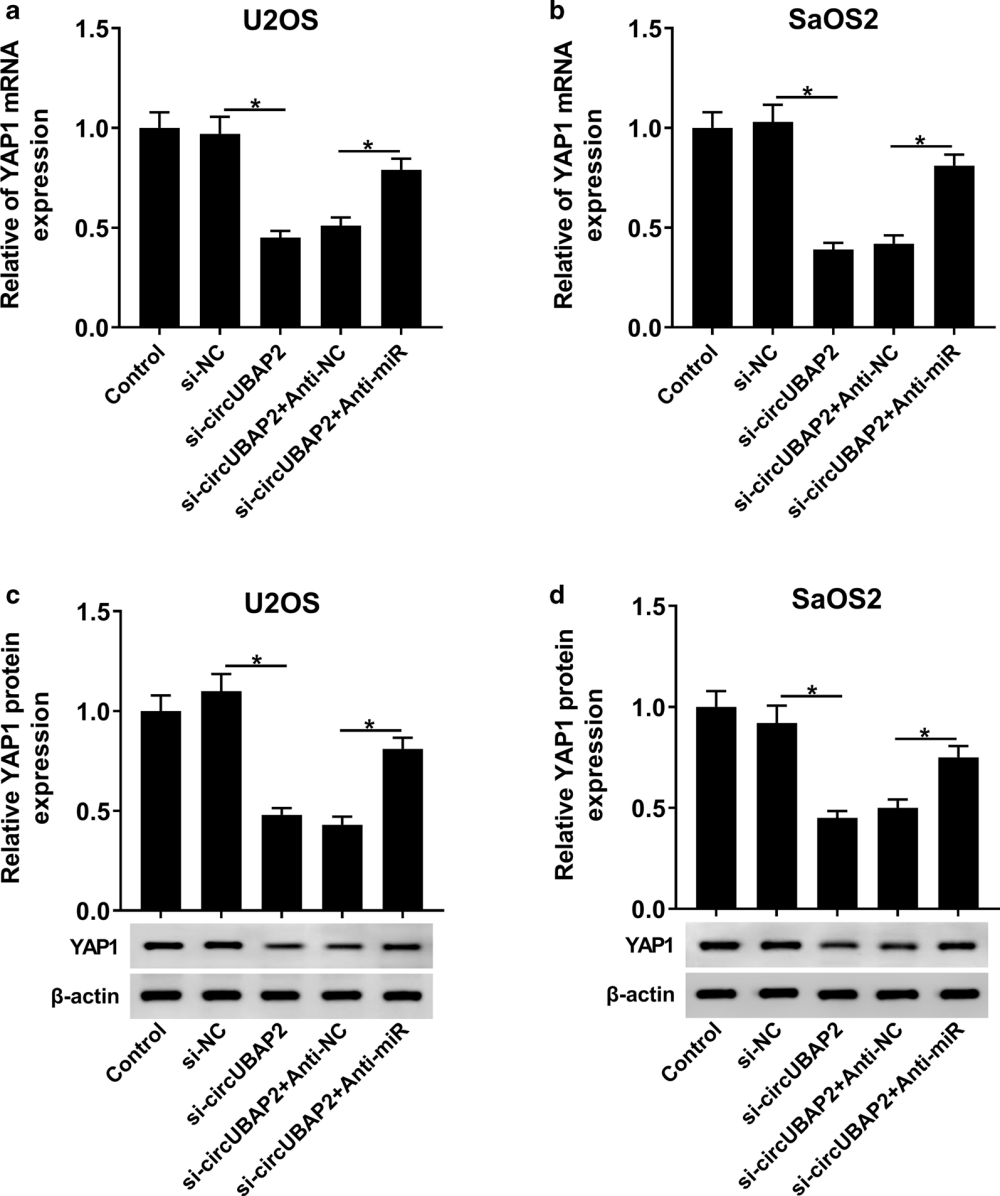
circUBAP2 regulated YAP1 by interacting with miR-641 in OS cells. **a**, **b** The mRNA level of YAP1 in Control and OS cells infected with si-circUBAP2, si-circUBAP2 + Anti-miR or corresponding controls was detected by qRT-PCR. **c**, **d** The protein level of YAP1 in Control and infected OS cells was measured by western blot. *P < 0.05

From results above, we knew that circUBAP2 interacted with miR-641 and miR-641 could target the 3′UTR of YAP1, which aroused our interest to explore the underlying regulatory mechanism among them. U2OS and SaOS2 cells were first transfected with si-circUBAP2, si-circUBAP2 + Anti-miR or matched controls. Afterwards, the mRNA and protein levels of YAP1 in Control and transfected OS cells were measured and the results showed that YAP1 was significantly downregulated in si-circUBAP2 group, while its level was inverted after the transfection with Anti-miR (Fig. 7a–d). Taken together, these results suggested that circUBAP2 might function as an endogenous sponge to interact with miR-641 and its silencing upregulated miR-641, thus inhibiting the translation of YAP1 in OS cells.

## Discussion

OS is a growing threat to young people, and patients with clinical metastasis have a low five-year survival rate [2]. Therefore, it is essential to find new molecular targets and investigate potential mechanisms, which will contribute to the development of more effective treatment for OS.

CircRNAs have been verified to regulate the progression of many cancers. Wang et al. found that circRHOT1 promoted hepatocellular carcinoma progression [7]. Li et al. reported that circ-DONSON boosted gastric cancer growth [8]. Ma et al. confirmed that circTADA2A facilitated osteosarcoma progression and metastasis [9]. To explore the function of circUBAP2, we checked its expression level and found that circUBAP2 was conspicuously upregulated in OS tissues and cells, which was in line with a previous report [10]. Further studies indicated that downregulation of circUBAP2 restrained proliferation and invasion of OS cells. In addition, circUBAP2 silencing inhibited the process of EMT, which was important for the initiation of metastasis [27]. All in all, these results demonstrated that circUBAP2 might act as an oncogene in the progression of OS.

Growing evidence has clarified the fact that circRNAs could serve as the sponges of miRNAs to function in many cancers [28, 29]. In this study, miR-641 was predicted to be a target of circUBAP2 and the interaction was verified by the dual-luciferase reporter assay. Previous studies showed that miR-641 served as a tumor suppressor in human lung cancer [17] and induced apoptosis of cervical cancer cells [18]. To study the function of miR-641, we checked its expression and found that miR-641 was apparently downregulated in OS tissues and cells, which was supported by Chen’s research [19]. In-depth research showed that miR-641 was negatively regulated by circUBAP2 in OS cells and miR-641 depletion boosted proliferation and invasion of OS cells and promoted the process of EMT. To sum up, these results illustrated that circUBAP2 might serve as the sponge of miR-641, which functioned as a tumor suppressor in OS progression.

To deeply explore the mechanism of miR-641 in OS, its target genes were predicted and YAP1was confirmed to be a target of miR-641. We then checked the protein level of YAP1 in OS cells and found that miR-641 negatively regulated the expression of YAP1. Further studies indicated that overexpression of YAP1 transposed miR-641-mediated repressed impacts on proliferation and invasion of OS cells. Meanwhile, upregulation of YAP1 also rescued the suppressed effect of miR-641 on the process of EMT. To investigate the regulatory relationship among circUBAP2, miR-641 and YAP1, the mRNA and protein levels of YAP1 in OS cells transfected with si-circUBAP2 or si-circUBAP2 + Anti-miR, as well as corresponding controls, were measured. The results indicated that circUBAP2 silencing significantly reduced the expression of YAP1 in OS cells, whereas the level of YAP1was upregulated following the infection with Anti-miR. Taken together, these results suggested that circUBAP2 might act as a sponge, interacting with and downregulating miR-641, thus damaging the translational inhibition of YAP1, ultimately promoting proliferation and invasion of OS cells.

## Conclusion

In conclusion, our research disclosed that circUBAP2 was strikingly upregulated in OS tissues and cells. Also, circUBAP2 could regulate proliferation and invasion of OS cells by miR-641/YAP1 axis. This novel mechanism may provide new effective therapeutic methods for OS.

## Supplementary information

**Additional file 1: Figure S1.** circUBAP2 knockdown downregulated the expression of C3aR and ICAM-1 in OS cells. (A and B) The protein level of C3aR and ICAM-1 in Control and OS cells transfetced with si-NC, or si-circUBAP2 was detected by western blot. *P < 0.05.

## Data Availability

All data generated or analysed during this study are included in this published article.

## Abbreviations

OS: Osteosarcoma
CircRNAs: Circular RNAs
qRT-PCR: Quantitative real-time polymerase chain reaction
CCK-8: Cell Counting Kit-8
CCK-8: Cell Counting Kit-8
EMT: Epithelial–mesenchymal transition
3′UTR: 3′-untranslated region
MiRNAs: MicroRNAs
mRNA: Messenger RNA
