# Supplementary figures and images for: Circular RNA circUBAP2 regulates proliferation and invasion of osteosarcoma cells through miR-641/YAP1 axis
Source: Cancer Cell Int. 2020 Jun 8;20:223. doi: 10.1186/s12935-020-01318-4 (PMC7282129; doi:10.1186/s12935-020-01318-4)

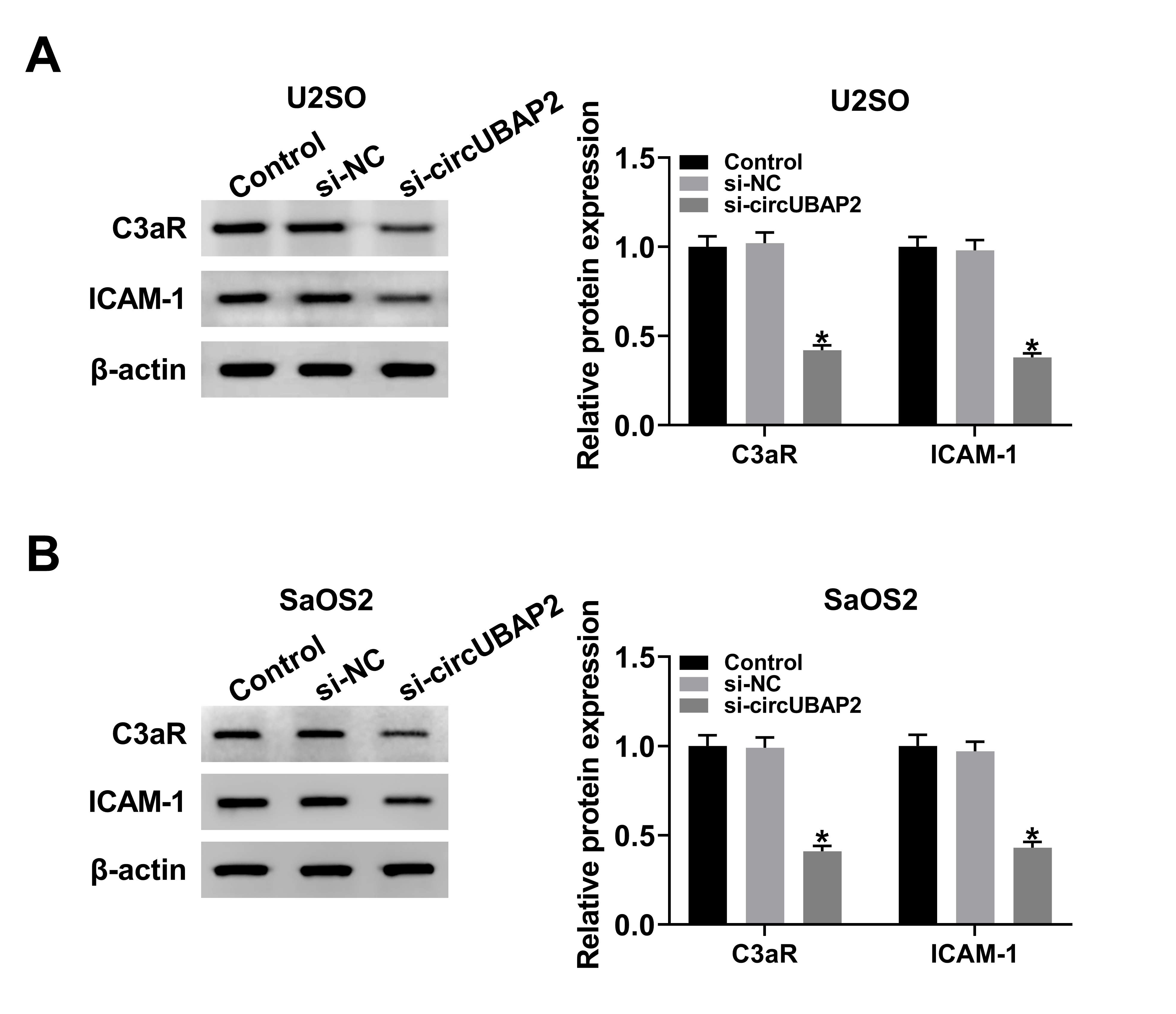

Supplement: Supplementary file 1 — Additional file 1: Figure S1. circUBAP2 knockdown downregulated the expression of C3aR and ICAM-1 in OS cells. (A and B) The protein level of C3aR and ICAM-1 in Control and OS cells transfetced with si-NC, or si-circUBAP2 was detected by western blot. *P < 0.05. [file 12935_2020_1318_MOESM1_ESM.tif]
